# Principal Components for Practice‐Oriented Measurement of Running Technique: A Proof‐Of‐Concept Study

**DOI:** 10.1002/ejsc.70004

**Published:** 2025-06-27

**Authors:** Daniel Debertin, Julia Kiebacher, Martin Zhang, Peter Federolf

**Affiliations:** ^1^ Department of Sport Science University of Innsbruck Innsbruck Austria

**Keywords:** optical motion capture, principal component analysis, running kinematics, technique measures

## Abstract

This study aims to construct valid and practically applicable running technique measures using principal component analysis (PCA). We hypothesized that data‐driven principal movements (PMs), derived from deliberately instructed opposite technique variations, would significantly distinguish these variations and could serve as quantitative measures of running technique as described by practitioners. 20 experienced runners were instructed to vary 14 distinct running technique elements into two opposing directions (e.g., forward and backward lean for a technique element representing horizontal movements). Elements and their variations were selected based on visual descriptions from practitioners found in running literature. Kinematic data were collected on a treadmill using optical motion capture and analyzed using a PCA‐based approach to determine running‐specific technique measures per technique element. By combining trials with opposing technique variations, variance in the data was purposefully produced, which in turn caused the resultant principal movements to align with the intended technique element. For all of the 14 technique elements, a valid measure—in the sense that the inputted opposite variations were significantly distinguishable within this measure—could be constructed. The measures could further be applied to the habitual running technique of the group of tested runners. The results of this study demonstrate the construct validity and applicability of the presented approach to measure running technique. This method can provide runners and coaches with valuable feedback and will enable future studies to investigate running technique, quantified through practice‐informed measures, in the context of performance, injury risk, or adaptations to equipment.

## Summary

- The presented approach turns the descriptive terms of running technique (e.g., forward lean, knee lift, and pelvic rotation) into quantifiable measures.
- As the resultant measures are practice‐informed, they become intuitively clear for practitioners. This facilitates communication between scientists and practitioners when analyzing running technique and implementing potentially beneficial technique elements in daily training routines.
- The developed technique measures allow for comparisons of habitual running against different technique variations. These comparisons can serve as a reference to further examine the effects of technique variations on performance‐related variables, injury risks, or adaptations to running equipment.

## 1 Introduction

Physical activity is an important factor for a healthy lifestyle (Fuchs 2003). Globally, running is one of the most popular forms of physical activity (Scheerder et al. 2015), due to its natural form of human locomotion and minimal required resources (Bramble and Lieberman 2004). Adopting a suitable “running technique” is often pursued with the aim of improving economy (Folland et al. 2017; Moore 2016; Williams and Cavanagh 1987) and potentially reducing load‐related risk factors associated with injury (Dillon et al. 2023; Van Hooren et al. 2024), although the direct relationship between specific techniques and injury prevention remains complex and controversial in the literature (Jauhiainen et al. 2020; Peterson et al. 2022). Technique refers to the individual multisegment movement patterns that an athlete employs in the specific sport discipline (Lees 2002). Practically, coaches usually tend to observe an athlete's technique by focusing on specific elements, such as forward or backward leaning of the upper body. However, when analyzing running technique scientifically, these descriptions do not necessarily align with the usually investigated variables, such as discrete joint angles. This creates a demand for a comprehensive, quantitative, but also practice‐informed technique analysis, that is, valuable for both practitioners, such as athletes or coaches as well as scientists, who require objective data rather than qualitative descriptions when they investigate, for example, running economy and performance improvement, differences between runner groups, well‐being enhancement through running, or injury prevention (Elphinston 2008).

An “ideal” running technique is primarily associated with improved running economy defined as the metabolic cost of running at a given submaximal speed (Van Hooren et al. 2024). Although running technique is highly individual, evidence suggests that certain key techniques can positively influence running economy. Practical movement executions in this regard include maintaining a slight forward lean while running (Williams and Cavanagh 1987), low vertical oscillation of the body's center of mass, and an arm motion of smaller amplitude (Anderson 1996). Additional aspects of anatomically and functionally correct running technique encompass an upright pelvic and head posture, parallel alignment of leg axes, hip extension, pelvic stability, pronounced knee and heel lift, spinal alignment, and parallel arm swing with a 90° elbow angle (Larsen et al. 2019). Precise measurement and analysis of such running technique elements require their conversion into quantifiable variables through precise recording and computational analysis.

Variables for analyzing running technique primarily include spatiotemporal measures (e.g., stride length/frequency), discrete kinematics of trunk, upper, and lower limbs (joint angles and segmental accelerations), kinetic factors (ground reaction forces), or neuromuscular elements (Moore 2016; Van Hooren et al. 2024). Some studies tried to take a higher number of these variables into account to assess running coordination by an uncontrolled manifold (UCM) analysis (Möhler et al. 2020) or to extract the relevant single features by applying a principal component analysis (PCA) to joint angle waveforms (Jiang et al. 2023; Osis et al. 2014; Quan et al. 2021). However, PCA on kinematic data (Troje 2002) yields an alternative approach to identify patterns in the running movement and to reduce data dimensionality (Mohr et al. 2021). An advantage of this PCA‐based approach—extracting “principal movements” (PMs) that represent coordinated partial movements defined by the eigenvectors (principal components, PCs)—is its reliance on whole‐body segment positions rather than discrete kinematics (P. A. Federolf 2016). This allows for the integration of all available movement information and enables the analysis of correlated segmental movement patterns that constitute “technique” in sports (P. Federolf et al. 2014; Witte et al. 2010). This can be further extended in such a way that the resulting PMs align with practical movement executions or technique variations (Debertin et al. 2022), that are described by practitioners of the specific sport and consequently, can directly be interpreted by them.

The objective of the current study is to develop a valid and applicable method for objectively measuring running technique, focusing on how body actions (relative movements between body segments) affect the running motion. The measures should align with practical technique descriptions from practitioners. Therefore, the primary aim was to develop and establish the construct validity of quantifiable practitioner‐informed running technique measures using PCA. We hypothesized that PMs derived from deliberately instructed opposite technique variations would significantly distinguish these variations (I) and could be applied as measures to quantify running technique within the tested group of experienced runners (II).

## 2 Materials and Methods

### 2.1 Participants

A total of 20 participants (10 male and 10 female) were recruited to establish the running technique measures. These participants were experienced and well‐trained runners who met the following inclusion criteria: regular running training in the past two months (approximately three times per week for more than 30 min), minimum three years of running experience, and an age between 18 and 45 years. Exclusion criteria included current injuries or other issues that could affect their running. All participants were students at the University of Innsbruck and were informed about the study's background and goals before providing written consent. The average age of the participants was 25.7 (± 2.2) years, with an average height of 172.9 (± 6.6) cm and an average weight of 65.8 (± 8.4) kg. The study protocol had been reviewed and accepted by the University of Innsbruck’s Board for Ethical Questions in Research (certificate 102/2022).

### 2.2 Procedures

The project involved marker‐based recording of predefined running technique variations executed by experienced runners on a treadmill in a lab setting. Running movement was captured using retroreflective markers positioned on anatomical landmarks on all body segments of the participants according to the marker setup “Plug‐in Gait” (Vicon Motion Systems Ltd., Oxford, UK). All participants were asked to bring their own running shoes. To facilitate marker placement, male participants wore tight running shorts and female participants wore tight running shorts and sports bras. Before the measurements, each participant had the opportunity to warm up on the treadmill.

Fourteen distinct running technique elements were selected based on visual descriptions found in literature (Larsen et al. 2019). The runners demonstrated each technique element by performing two opposite variations per element (Table 1), which were instructed through verbal cues and live demonstrations by the study supervisors. The variations could reflect extreme postures or more habitual running techniques as long as a clear and distinguishable difference existed between them. In addition, each participant ran with their preferred running technique at the beginning and end of the in total 28 technique variation trials, resulting in two “habitual” trials per participant. Each trial was recorded for 15 s. Female participants ran at 2.22 m·s^−1^ (8 km·h^−1^) and male participants ran at 2.78 m·s^−1^ (10 km·h^−1^). Rather low speeds were chosen to facilitate a clear and consistent demonstration of the instructed techniques. Different speeds for the sex groups were chosen based on their distinct fitness levels as indicated by self‐reported running FTP.

### 2.3 Data Acquisition and Analysis

Participants' kinematic data of segment positions while running were collected using the Vicon MX Motion Capture System (Vicon Motion Systems Ltd., Oxford, UK). This system used a full‐body model with 39 markers (“Plug‐in Gait”), 10 cameras (8 Vicon Bonita 10 and 2 Vicon Vero v2.2 cameras), and a sampling rate of 250 Hz.

The 3D positional data (marker coordinates) obtained from the Vicon Nexus Software Version 2.12.1 (Vicon Motion Systems Ltd., Oxford, UK) were analyzed using a principal component‐based approach (Debertin et al. 2022; P. Federolf et al. 2014; Gløersen et al. 2018). The procedure was adapted for running technique analyses and involved the following preprocessing steps: (i) filtering data by a fourth order low‐pass Butterworth filter with a cutoff frequency of 10 Hz; (ii) transforming position data into a pelvis‐fixed reference system for participants; (iii) identifying and extracting 10 consecutive gait cycles per trial, from right foot strike to right foot strike, using the vertical foot position algorithm described by Fellin and colleagues, which detects foot strike based on the minimum vertical position of the heel marker (Fellin et al. 2010); (iv) centering data of each participant's recorded trial by subtracting the mean from the habitual running trials of this participant; and (v) normalizing marker coordinates by mean Euclidean distance (P. A. Federolf 2016; P. Federolf et al. 2013) and weighting of the body segment masses according to de Leva (1996).

To obtain a specific measure for each of the 14 running technique elements, we conducted a separate PCA for each element. Specifically, the input data matrix for each of these 14 PCAs consisted only of the preprocessed time‐series 3D positional data from all participants performing the two instructed opposite variations of the respective element (e.g., only data from the “forward lean” and “backward lean” trials to obtain a PC for the technique element of “horizontal movement” actions). This element‐wise approach was chosen to ensure that a resulting PM specifically captures the variance of the intended technique element, thus providing a targeted measure for it. The preprocessing steps and the PCAs themselves were conducted by means of an existing toolbox called “PManalyzer” (Haid et al. 2019), written in MATLAB (The MathWorks, Natick, Massachusetts, USA). PCA extracts correlated segment movement patterns mathematically, providing a new movement‐specific coordinate system defined by eigenvectors (PCs) of the covariance matrix. Each PC indicates a specific direction of deviation in body configuration from the mean posture and thus represents a PM. Characteristics of each PM along a PC can be assessed by examining principal positions (PPs):

(1) $$PP_{k}\left(t\right)=\frac{p\left(t\right)\;-\;{\bar{p}}_{\text{standard}}}{d_{\text{norm}}}\;\cdot \;\omega \;\cdot \;PC_{k},$$

with the order of the component *k*, the posture vector *p(t)* (timesteps x dim, dim: 39·3 marker coordinates in 3D), the mean posture vector from the habitual running trials ${\bar{p}}_{\text{standard}}$ (1 x dim), the mean Euclidean distance *d*_norm_, the diagonal weight matrix *ω* (dim x dim), and the principal component vector *PC*_*k*_ (dim x 1). The *PP*_*k*_ *(* *t* *)* waveforms can also be referred to as PC scores and measure participants' movements expressed through the PMs (P. A. Federolf 2016). The first‐order PM explains the most variability in the dataset; further lower‐order PMs (e.g., PM_2_, PM_3_, …) also contain main movement patterns characteristic for running; higher‐order PMs refer to submovements that capture less variance in the data. Instructing participants to perform opposing technique elements and combining these trials into one input matrix for the PCA purposefully increases the variance in the dataset. Consequently, the lower‐order PMs aligned with the given posture changes that had been caused by technical instruction. One or a combination of several of the lower‐order PC vectors then closely approximate the underlying technique element. Projecting running data onto this/these vector(s) then yields scores/PP‐waveforms that quantify how much at a given time point the athlete's posture aligned with the underlying technique element, facilitating a quantitative technique evaluation.

The postprocessing steps were self‐coded in MATLAB and involved selecting the order of the PM or a combination of PMs, which express the technique element movement. Thereby, the first 15 PMs were visualized as stick figure animations in the original 3D space and the PM that clearly involved segmental movements associated with the respective technique element of interest was manually selected based on visual inspection. In cases where multiple PMs reflected the relevant technique, we combined them by summing the respective PM matrices. For these PM selections, each corresponding PP waveform was time‐normalized per cycle through interpolation and the mean of the 10 cycles was calculated per participant. PP waveforms for the habitual trials were not used as inputs for the PCA but were projected onto the derived PCs using Formula (1). The mean of the cycles from the two habitual trials was taken for further statistical analyses.

### 2.4 Statistics

For each of the 14 PM selections, that express a running technique element, the three corresponding PP waveforms (variation (+) and (−) of the respective element and the habitual running technique) of all participants were analyzed using statistical parametric mapping (SPM) with a repeated measures ANOVA design (Pataky et al. 2013) and a significance level of *α* = 0.05. If significant differences were found, post hoc SPM‐based *t*‐tests with a Bonferroni correction were conducted. For the first purpose of this study, to analyze the construct validity of the newly defined technique measures in the shape of the associated PMs, post hoc tests between the two opposite variations were used. For the second purpose, to apply the measures and to quantitatively analyze the running technique, post hoc results between the two opposite variations with regard to the habitual running PP waveform were investigated.

## 3 Results

### 3.1 PMs for 14 Running Technique Elements

**FIGURE 1:**
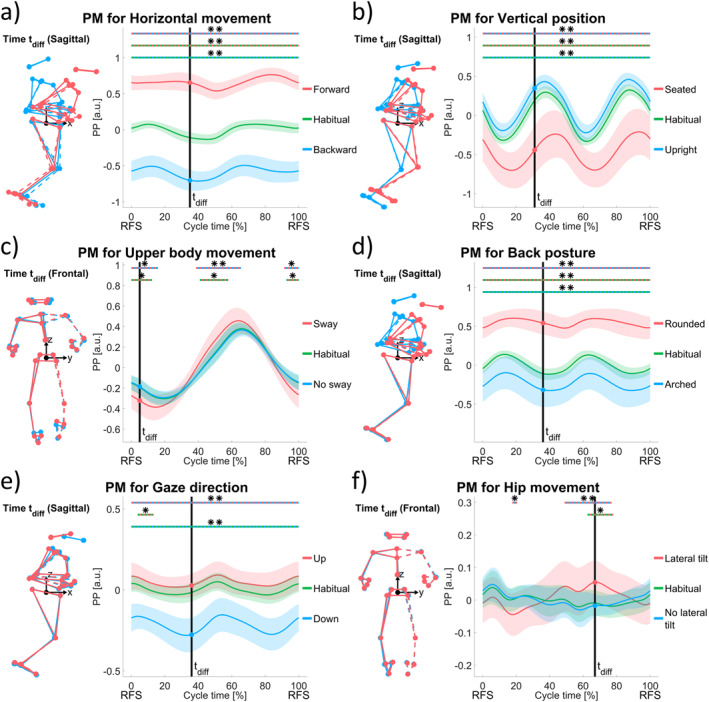
Six panels of principal movements (PMs) expressing running technique elements: (a) Horizontal movement, (b) vertical position, (c) upper body movement, (d) back posture, (e) gaze direction, and (f) hip movement. On the right of each panel, averaged principal position (PP) waveforms are shown for two opposite variations (= input for PCA, red and blue lines with shaded areas representing standard deviations) within the respective technique element and the habitual running trials projected onto the PM (green line with standard deviation area) of experienced runners (*N* = 20). For each runner, waveforms were averaged from 10 normalized consecutive running cycles (from right foot strike (RFS) to right foot strike) beforehand. Cycle times of statistically significant differences from three post hoc tests after a statistical parametric mapping (SPM) with repeated measures ANOVA design (Pataky et al. 2013) are marked as colored horizontal dashed lines (top line: variation (+) versus variation (−); middle line: habitual versus variation (+); and lower line: habitual versus variation (−)). Significant differences are indicated by * (*p* < 0.05) and ** (*p* < 0.001). On the left of each panel, configurations of the corresponding extracted movement are plotted for the two opposite variations at the time point, where the waveforms of these opposites differed the most (vertical black line). Configurations also illustrate the means over all participants.

**FIGURE 2:**
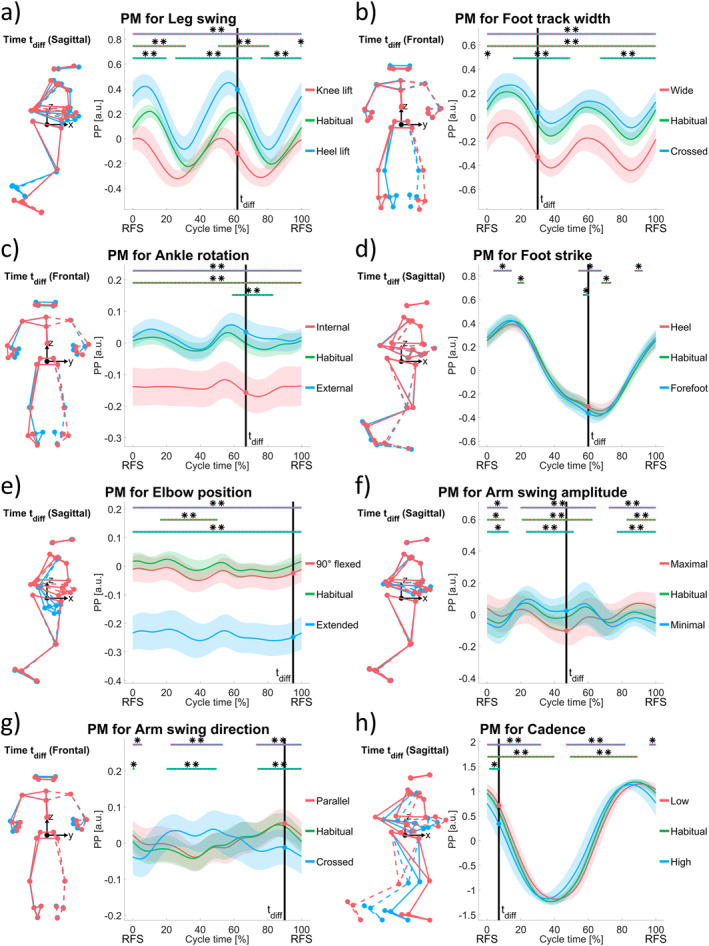
Eight panels of principal movements (PMs) expressing running technique elements: (a) Leg swing, (b) foot track width, (c) ankle rotation, (d) foot strike, (e) elbow position, (f) arm swing amplitude, (g) arm swing direction, and (h) cadence. Format and labeling of each panel are the same as in Figure 1.

Figures 1 and 2 show the 14 selected PMs (videos of animated stick figure configurations are included in the Supporting Information S1 section) for the different running technique elements based on the aforementioned procedure. SPM results are marked by horizontal lines above the PP waveforms and are provided in detail in Table 1. At first, the technique element measures are tested for their construct validity by comparing the two opposite variation waveforms (blue and red lines).

**TABLE 1:** Descriptions of 14 running technique elements and their two opposite variation forms. For each technique element, the orders of the selected principal movements (PMs) associated with this element as a result from an element‐wise principal component analysis (PCA) is stated. Additionally, the results of three post hoc comparisons (between the principal position (PP) waveforms of two opposite variations and the habitual running technique along the resultant PM) after a statistical parametric mapping (SPM) analysis with repeated measures ANOVA design (Pataky et al. 2013) are indicated. These involve the cycle parts where significant differences occurred (clusters) together with their *p*‐values and the overall duration of the clusters relative to the entire running cycle. Percentage values are rounded to whole numbers after the total sum was calculated.

Technique element	Technique variation (+)	Technique variation (−)	Principal movement (PM)	Post hoc variation (+) versus (−)			Post hoc variation (+) versus habitual technique			Post hoc variation (−) versus habitual technique		
				Cluster [%]	*p‐*value	Sum [%]	Cluster [%]	*p‐*value	Sum [%]	Cluster [%]	*p‐*value	Sum [%]
Horizontal movement	Forward lean	Backward lean	2	0–100	< 0.001	100	0–100	< 0.001	100	0–100	< 0.001	100
Vertical position	High hip position (“upright”)	Low hip position (“seated”)	2	0–100	< 0.001	100	0–100	< 0.001	100	0–100	< 0.001	100
Upper body movement	Sway from side to side	No sway (central upper body position)	2	0–16	0.002	51	0–12	0.004	37			0
				39–65	< 0.001		41–58	0.001				
				91–100	0.009		93–100	0.01				
Back posture	Rounded back (“hunchback”)	Arched back	2	0–100	< 0.001	100	0–100	< 0.001	100	0–100	< 0.001	100
Gaze direction	Look up	Look down	4	0–100	< 0.001	100	4–13	0.016	10	0–100	< 0.001	100
Hip movement	Lateral tilt (unstable pelvis)	No lateral tilt (stable pelvis)	11	18–21	0.015	31	63–78	0.001	15			0
				49–77	< 0.001							
Leg swing	High knee lift	High heel lift	4 + 5	0–100	< 0.001	100	0–31	< 0.001	62	0–20	< 0.001	90
							51–81	< 0.001		25–71	< 0.001	
							99–100	0.017		76–100	< 0.001	
Foot track width	Wide	Crossed	4	0–100	< 0.001	100	0–100	< 0.001	100	0–1	0.017	67
										15–49	< 0.001	
										67–100	< 0.001	
Ankle rotation	Internal rotation	External rotation	7	0–100	< 0.001	100	0–100	< 0.001	100	59–83	< 0.001	24
Foot strike	Forefoot strike	Heel strike	3	4–15	0.004	29	18–22	0.014	10	57–61	0.014	4
				54–68	0.001		68–74	0.011				
				87–92	0.013							
Elbow position	90° flexed elbows	Fully extended elbows	4	0–100	< 0.001	100	16–50	< 0.001	34	0–100	< 0.001	100
Arm swing amplitude	Arms swing maximally	Arms swing minimally	6	0–12	0.003	85	0–10	0.004	70	0–13	0.001	64
				20–65	< 0.001		21–62	< 0.001		23–51	< 0.001	
				72–100	< 0.001		83–100	< 0.001		77–100	< 0.001	
Arm swing direction	Parallel arm swing	Crossed arm swing	11	0–6	0.015	63			0	0–1	0.017	57
				22–53	< 0.001					20–50	< 0.001	
				73–100	< 0.001					74–100	< 0.001	
Cadence	Low cadence	High cadence	1	0–32	< 0.001	71	0–40	< 0.001	80	1–7	0.008	6
				47–82	< 0.001		49–89	< 0.001				
				96–100	0.014							

Horizontal movement variations (forward and backward lean) were clearly distinguishable in their associated PM (Figure 1a, Video S1). Statistically, the two opposite technique variations were separable in 100% of the cycle (*p* < 0.001).

Upright versus a seated running style, that is, the vertical positioning, was also clearly discernible within its associated PM (Figure 1b, Video S2). The two corresponding waveforms were significantly different along the whole cycle (0%–100%, *p* < 0.001). The deepest vertical position (lower PP values) was observed during stance phase for all trials. A seated running style had a high standard deviation across participants.

For the task to emphasize lateral upper body sway (Figure 1c, Video S3), the upper body swung more to the right (lower PP values) during the stance phase of the right leg and more to the left (higher PP values) during the stance phase of the left leg than for the opposing task to avoid lateral sway. Apart from the crossing points of the waveforms, they were statistically separable (0%–16%, *p* = 0.002; 39%–65%, *p* < 0.001; and 91%–100%, *p* = 0.009).

Different back postures were consistently reflected in different waveform shapes over the whole cycle (0%–100%, *p* < 0.001) within the resultant PM (Figure 1d, Video S4). A rounded back occurred on lower levels and an arched back on higher levels of the PP axis.

Similarly, a PM for different gaze directions (Figure 1e, Video S5) indicated consistently higher PP values for upwards than for downwards direction (0%–100%, *p* < 0.001).

Hip movement strategies (lateral tilt and no lateral tilt) showed cycle parts of significant differences mainly during stance phases (18%–21%, *p* = 0.015; 49%–77%, *p* < 0.001) within the associated PM (Figure 1f, Video S6). Standard deviations areas were greater for the lateral tilt instruction.

Leg swing variations (pronounced knee lift vs. pronounced heel lift) differed significantly for 100% of the cycle (*p* < 0.001) in the PM, that mainly involves lower limb movements (Figure 2a, Video S7).

A PM for foot track width could clearly be detected (Figure 2b, Video S8). Again, 100% of the cycle differed significantly between the two opposite variations (*p* < 0.001). The feet were closer together during stance phase (highest PP values) in either case.

Ankle rotation variations were significantly distinguishable through a PM as well (Figure 2c, Video S9). The whole cycle (0%–100%, *p* < 0.001) differed between opposite techniques of internally and externally rotated ankles.

Foot strike (heel vs. forefoot strike) mostly influenced a PM (Figure 2d, Video S10), characterized by slightly different toe positions (more to the front for the forefoot strike) within the stance phase. As the focus for this technique element was mainly on the stance phases, cycle parts of statistically significant differences received from SPM were smaller (4%–15%, *p* = 0.004; 54%–68%, *p* = 0.001; and 87%–92%, *p* = 0.013).

A sagittal elbow angle of 90° led to an almost constant PP value over the whole cycle in the respective PM (Figure 2e, Video S11), whereas PP values of fully extended elbows were significantly lower over the whole cycle (0%–100%, *p* < 0.001).

The arm swing amplitude specific PM (Figure 2f, Video S12) as a result from maximal and minimal arm swing variations, mainly depicted arm movements in the sagittal direction. Around right foot contact, the left arm was positioned further forward and the right arm further backward for the maximal than for the minimal arm swing instruction. Differences were observable within the majority of the cycle time (0%–12%, *p* = 0.003; 20%–65%, *p* < 0.001; and 72%–100%, *p* < 0.001).

The arm swing direction specific PM (Figure 2g, Video S13) mainly implied arm movements in the frontal plane. Around right foot contact, the left arm was laterally closer to the pelvis center (lower PP values) for the crossed than the parallel arm swing instruction and vice versa around left foot contact times. As the two waveforms crossed each other, the SPM cluster covered a total of 63% of the cycle (0%–6%, *p* = 0.015; 22%–53%, *p* < 0.001; and 73%–100%, *p* < 0.001).

Cadence variations mainly influenced a PM involving leg and arm swing movements together (Figure 2h, Video S14) with higher amplitudes for the low cadence instruction (0%–32%, *p* < 0.001; 47%–82%, *p* < 0.001; and 96%–100%, *p* = 0.014).

### 3.2 Habitual Running Technique Evaluated Through PM Measures

As mentioned in the methods section, the habitual running trials without specific instructions were projected onto the obtained PMs afterward (green waveforms in Figures 1 and 2), that is, they did not influence the PMs. Following results are based on the SPM post hoc tests of the habitual running waveforms against either of the two variations within the respective technique element measure.

For the horizontal movement (Figure 1a), the vertical position (Figure 1b), the back posture (Figure 1d), the leg swing (Figure 2a), the foot track width (Figure 2b), and the arm swing amplitude (Figure 2f) PMs the habitual technique was significantly different compared to the two opposite variations over a large proportion of the whole cycle. While the PP waveform of the habitual technique was positioned in the middle between the forward and backward lean trials as well as between pronounced knee and heel lift, it was closer to an upright vertical position, a straighter back posture, a narrower foot track width, and a minimal arm swing amplitude. No, or almost no, significant differences of the habitual technique were found in comparison to the tasks with no upper body sway (Figure 1c), upward gaze direction (Figure 1e), no lateral tilt of the hip (Figure 1f), external ankle rotation (Figure 2c), heel and forefoot strike (Figure 2d), 90° flexed elbows (Figure 2e), parallel arm swing (Figure 2g), and high cadence (Figure 2h).

## 4 Discussion

The purposes of this study were twofold. First, construct validity of the quantifiable running technique measures aligning with practice‐informed segmental movements was shown. Second, the running technique of 20 experienced runners could then be evaluated by applying the specific technique element measures.

### 4.1 Quantifiable Measures for 14 Distinct Running Technique Elements

For each of the 14 distinct running technique elements, the two respective variations, on which an element‐wise PCA was conducted, showed at least some parts of the gait cycle where SPM‐based statistically significant differences were found in their associated element‐specific PM. Particularly, different horizontal movements, vertical positions, back postures, gaze directions, leg swing strategies, foot track widths, ankle rotations, and elbow positions resulted in clearly separable PP waveforms (during 100% of the cycle). This was expected, as these movements allow for a wide variation range along the whole cycle, where the given instructions were realizable to a great and clear extent. Meeting these expectations and statistically confirming them proves the construct validity of the defined technique measures. Different upper body and hip movement strategies as well as different foot strike patterns, arm swing amplitudes, arm swing directions, and cadences resulted in parts of the cycle where statistically significant differences were observed in the respective technique‐specific PM. This can be mainly attributed to crossing PP waveforms. In the actual time phases, where different movement techniques were expected, these differences could still be confirmed statistically.

Consequently, a PCA‐based method for technique analyses was successfully transferred and adapted to running.

Compared to conventional technique analyses focusing on discrete spatiotemporal or kinematic variables (e.g., contact time, stride length, and joint angles at specific events) (Moore 2016; Van Hooren et al. 2024), the PCA‐based approach presented here offers several advantages. The approach inherently enables the quantification of coordinated multisegmental movement patterns across the entire gait cycle, rather than limiting the analysis to isolated events. Importantly, by aligning the resulting PMs with practitioner‐defined technique elements through the use of instructed variations, the method generates quantifiable measures that are presumed to be more intuitively interpretable by practitioners. This, in turn, has the potential to bridge the gap between scientific technique analysis and practical application in coaching or rehabilitation settings.

Compared to P. Federolf et al. (2014), Gløersen et al. (2018), and Mohr et al. (2021), the current approach benefits from applying PCA on purposefully instructed technique variations rather than just the unvaried movements. This reveals a new perspective that makes certain technique variations distinguishable and readable on a scale (PC score) for any time point over the gait cycle. This new perspective with the advantage of being directly based on practical movements makes animations or stick figure representations of the technique elements equally vivid and intuitively clear for athletes and coaches.

Compared to Debertin et al. (2022), several PCAs were conducted element‐wise to better align each technique element with one desired specific measure.

### 4.2 Applicability of PCA‐Based Running Technique Measures

The running technique measures constructed from the technique “opposites” could be applied on the runners' individual techniques, recorded without any technique instructions. The habitual running technique fell between the two opposite technique variations for the horizontal movement, vertical position, back posture, leg swing, foot track width, and arm swing amplitude element. In some cases, the habitual technique waveform was closer to either of the variations, for example, a more upright than seated vertical position, a more arched than rounded back, a more crossed than wide foot track width, and a more minimal than maximal arm swing amplitude. For other running technique elements, the habitual technique waveform almost coincided with either of the variations, for example, no sway of the upper body and no lateral tilt of the hip as well as upward gaze, external ankle rotation, 90° flexed elbows, and parallel arm swing direction. These findings can then be regarded as the running technique of the tested group of runners. As all the participants were experienced runners, some findings of their technique correspond to the “ideal” running technique previously reported in literature, for example, an arm motion of smaller amplitude (Anderson 1996) or an upright pelvic and head posture (Larsen et al. 2019). Others might reveal new insights into how the runners realized the technique practically, for example, through a stable upper body and hip position. A slight constant forward lean, which is considered economically advantageous (Williams and Cavanagh 1987), cannot be monitored, as the PCA input data were based on the mean posture from the habitual running trials (step iv in the methods section). However, the PM selected for horizontal movements still represents movements in this direction, allowing to analyze a forward or backward lean during different phases of the cycle. Different foot strike patterns of the participants were statistically distinguishable within the stance phases. At touchdown, the mean waveform of habitual running showed a tendency toward heel striking, making it interpretable as rearfoot strike, which is consistent with the comfortable running speeds at which the trials were performed (Van den Berghe, Warlop, et al. 2022). However, the stick‐figure visualizations do not make the foot strike techniques immediately recognizable, so that the practical application remains questionable for this technique element measure.

In summary for all other measures, plausibility and their feasibility for application could be confirmed. The obtained technique measures, aligning with practice‐informed running technique descriptions and providing objective and quantitative criteria, can now be applied and utilized for evaluating, categorizing, and comparing techniques within an individual runner, between runners, or between groups (e.g., of different age, gender, and background) both in a performance and injury‐related context.

In practical performance‐related application, the proposed technique measures—presented in a visually simplified format, such as animated stick figures alongside quantifiable scores—could be used by coaches to provide targeted and comprehensible feedback to runners. For instance, deviations in a runner's “leg swing” score from the average of expert performers could be identified. Based on this information, a coach might recommend targeted interventions, such as knee lift exercises, and subsequently assess whether these exercises lead to measurable changes in the corresponding technique measure. Further analysis could then examine whether the observed changes in “leg swing” are associated with improvements in performance. Although such alignment with expert technique does not necessarily guarantee performance enhancement—given the inherent individuality of technique—it may serve as a valuable starting point in the pursuit of performance optimization. Our framework is designed to support this process by enabling the careful monitoring and evaluation of technique adaptations in a practical long‐term training context. We further postulate that the PMs defined by the technique variations create sensitive, quantifiable scales along which not only techniques of extreme cases (Van den Berghe, Breine, et al. 2022) but also more subtle ecologically valid variations—such as those arising from fatigue or adaptations to footwear—can be measured. Future work should explicitly test the sensitivity of these PMs to subtler variations induced by such factors.

In injury‐related applications, the proposed technique measures could be utilized to assess whether an athlete's preinjury running technique—assuming routine recordings are available—has been fully restored or whether deviations persist due to unconscious protective behaviors or compensations (e.g., in the “hip movement” component). This enables objective monitoring of rehabilitation progress over time. Additionally, future studies could build upon this approach to provide a new perspective on the currently inconclusive relationship between running technique and injury risk—for instance, by relating specific quantifiable score waveforms from the technique measures to joint loading or kinetics during running and examining whether these are associated with a higher risk of injury.

Naturally, the expertise and knowledge of coaches and clinicians, as well as the traditional assessment of discrete technique variables, will remain indispensable. With our approach, we aim to provide a complementary computer‐based method for technique evaluation that supports qualitative observations and discrete analyses. This has the potential to reduce observer bias and facilitate practical implementation in applied settings.

### 4.3 Limitations

The visual selection process of PMs representing the technique elements of interest inherently involves a subjective component. However, by statistically confirming significant differences between the two contrasting input techniques within the technique‐specific PMs we associated with the respective inputs, we aimed to demonstrate the construct validity of our visually selected PMs as quantifiable technique element measures.

Additionally, for some technique elements (mainly foot strike patterns), discrete analyses might show larger differences. In general, our PCA approach only offers a complementary technique assessment method.

Furthermore, due to the individuality of every participant, technique variations might be performed differently, for example resulting in higher standard deviations for the tasks of seated vertical position and lateral hip tilt. However, the resultant measures still reflect the underlying technique element and enable an assessment of the different movement realizations. Nevertheless, measuring more participants could account for the individual differences and also improve the generalizability and external validity of the technique measures.

Although normalization procedures (mean Euclidean distance and segment mass weighting) were applied to mitigate anthropometric influences, we acknowledge that residual effects of body size and shape differences between participants could potentially influence the resulting PMs. Linear temporal normalization to gait cycle percentage was implemented; however, absolute time differences between trials, particularly for variations in cadence, may cause SPM outcomes to reflect not only amplitude differences but also timing‐related effects, which should be interpreted with caution. More advanced methods, such as nonlinear time warping, could in the future be used to disentangle these effects (Pataky et al. 2022).

Finally, confounders, such as footwear or training background of the participants, were not considered.

## 5 Conclusion

The project reported here aimed to develop quantifiable running technique measures, based on practice‐informed qualitative technique descriptions. The results presented demonstrate the suitability of the conceptual approach for the purpose of quantitatively evaluating running technique. The PCA‐based technique measures can be applied by researchers to both recreational and professional athletes to gain more insights into their running, for example, in comparison to others, or into how their running technique adapts to changes in environment or equipment. Key directions for future research include: (1) investigating potential correlations between specific PC scores and running economy, performance‐related variables, and injury risk and (2) quantifying adaptations in these technique measures in response to different footwear, fatigue protocols, or targeted training and rehabilitation interventions.

## Ethics Statement

The study protocol was approved by the Board for Ethical Questions in Science of the University of Innsbruck (certificate 102/2022) and followed the principles from the Declaration of Helsinki.

## Conflicts of Interest

The authors declare no conflicts of interest.

## Supporting information

Supporting Information S1

Video S1

Video S2

Video S3

Video S4

Video S5

Video S6

Video S7

Video S8

Video S9

Video S10

Video S11

Video S12

Video S13

Video S14

## Data Availability

The data supporting the findings of this study—specifically, the principal component vector data representing distinct technique elements—are available from the corresponding author upon request. The self‐coded scripts used for analysis are not publicly available but may be shared upon request for academic purposes. The research group plans to further develop the MATLAB toolbox PManalyzer (available at: https://www.uibk.ac.at/de/isw/forschung/neurophysiology‐of‐exercise/pm_analyzer/), with the aim of facilitating guided application of the technique analysis algorithms used in this study.
