# Supplementary material for: Principal Components for Practice‐Oriented Measurement of Running Technique: A Proof‐Of‐Concept Study
Source: Eur J Sport Sci. 2025 Jun 27;25(7):e70004. doi: 10.1002/ejsc.70004 (PMC12203835; doi:10.1002/ejsc.70004)
Supplement: Supplementary file 1 — Supporting Information S1 [file EJSC-25-e70004-s005.docx]

# Supporting Information - Video captions

**Video S1: Horizontal movement.** Time-dependent stick-figure animations of running with forward and backward lean within a principal movement (PM) that expresses horizontal movement. On the right, averaged principal position (PP) waveforms are shown for two opposite variations (= input for PCA, red and blue lines with shaded areas representing standard deviations) within the respective technique element and the habitual running trials projected onto the PM (green line with standard deviation area) of experienced runners (*N* = 20). For each runner, waveforms were averaged from 10 time-normalized consecutive running cycles (from right foot strike (RFS) to right foot strike) beforehand. Cycle times of statistically significant differences from three post-hoc tests after a statistical parametric mapping (SPM) with repeated measures ANOVA design are marked as colored horizontal dashed lines (top line: variation (+) vs. variation (-); middle line: habitual vs. variation (+); lower line: habitual vs. variation (-)). Significant differences are indicated by * (*p* < .05) and ** (*p* < .001). On the left, configurations of the corresponding extracted movement are animated for the two opposite variations over the time-normalized running cycle (vertical black line). Configurations also illustrate the means over all participants.

**Video S2: Vertical position.** Time-dependent stick-figure animations of running with an upright and seated posture within a principal movement (PM) that expresses vertical position. Format and labeling are the same as in Video S1.

**Video S3: Upper body movement.** Time-dependent stick-figure animations of running with and without upper body sway within a principal movement (PM) that expresses upper body movement. Format and labeling are the same as in Video S1.

**Video S4: Back posture.** Time-dependent stick-figure animations of running with a rounded an arched back within a principal movement (PM) that expresses back posture. Format and labeling are the same as in Video S1.

**Video S5: Gaze direction.** Time-dependent stick-figure animations of running with an upward and downward gaze within a principal movement (PM) that expresses gaze direction. Format and labeling are the same as in Video S1.

**Video S6: Hip movement.** Time-dependent stick-figure animations of running with and without lateral hip tilt within a principal movement (PM) that expresses hip movement. Format and labeling are the same as in Video S1.

**Video S7: Leg swing.** Time-dependent stick-figure animations of running with high knee and high heel lift within a principal movement (PM) that expresses leg swing strategies. Format and labeling are the same as in Video S1.

**Video S8: Foot track width.** Time-dependent stick-figure animations of running with wide and crossed track width within a principal movement (PM) that expresses foot track width. Format and labeling are the same as in Video S1.

**Video S9: Ankle rotation.** Time-dependent stick-figure animations of running with internally and externally rotated ankles within a principal movement (PM) that expresses ankle rotation. Format and labeling are the same as in Video S1.

**Video S10: Foot strike.** Time-dependent stick-figure animations of running with forefoot strike and heel strike within a principal movement (PM) that expresses foot strike patterns. Format and labeling are the same as in Video S1.

**Video S11: Elbow position.** Time-dependent stick-figure animations of running with 90° flexed and fully extended elbows within a principal movement (PM) that expresses elbow position. Format and labeling are the same as in Video S1.

**Video S12: Arm swing amplitude.** Time-dependent stick-figure animations of running with maximum and minimum arm swing within a principal movement (PM) that expresses arm swing techniques. Format and labeling are the same as in Video S1.

**Video S13: Arm swing direction.** Time-dependent stick-figure animations of running with parallel and crossed arm swing within a principal movement (PM) that expresses arm swing direction techniques. Format and labeling are the same as in Video S1.

**Video S14: Cadence.** Time-dependent stick-figure animations of running with low and high cadence within a principal movement (PM) that expresses cadence. Format and labeling are the same as in Video S1.
